# Perinatal and postnatal exposure to phthalates and early neurodevelopment at 6 months in healthy infants born at term

**DOI:** 10.3389/fendo.2023.1172743

**Published:** 2023-05-24

**Authors:** Laura Lucaccioni, Lucia Palandri, Erica Passini, Viola Trevisani, Filippo Calandra Buonaura, Natascia Bertoncelli, Giovanna Talucci, Angela Ferrari, Eleonora Ferrari, Barbara Predieri, Fabio Facchinetti, Lorenzo Iughetti, Elena Righi

**Affiliations:** ^1^ Pediatric Unit, Department of Medical and Surgical Sciences of the Mother, Children and Adults, University of Modena and Reggio Emilia, Modena, Italy; ^2^ Department of Biomedical, Metabolic and Neural Sciences, University of Modena and Reggio Emilia, Modena, Italy; ^3^ Clinical and Experimental Medicine PhD Program, University of Modena and Reggio Emilia, Modena, Italy; ^4^ Post graduate School of Pediatrics, Department of Medical and Surgical Sciences of the Mother, Children and Adults, University of Modena and Reggio Emilia, Modena, Italy; ^5^ School of Medicine, University of Modena and Reggio Emilia, Modena, Italy; ^6^ Neonatology Unit, Department of Medical and Surgical Sciences of the Mother, Children and Adults, University of Modena and Reggio Emilia, Modena, Italy; ^7^ Unit of Obstetrics and Gynecology, Department of Medical and Surgical Sciences of the Mother, Children and Adults, University of Modena and Reggio Emilia, Modena, Italy

**Keywords:** neurodevelopment, phthalates, perinatal exposure, endocrine disruptors, infants, newborns, Griffiths development scales

## Abstract

**Background:**

Phthalates are non-persistent chemicals largely used as plasticizers and considered ubiquitous pollutants with endocrine disrupting activity. The exposure during sensible temporal windows as pregnancy and early childhood, may influence physiological neurodevelopment.

**Aims and Scope:**

The aim of this study is to analyze the relationship between the urinary levels of phthalate metabolites in newborn and infants and the global development measured by the Griffiths Scales of Children Development (GSCD) at six months.

**Methods:**

Longitudinal cohort study in healthy Italian term newborn and their mothers from birth to the first 6 months of life. Urine samples were collected at respectively 0 (T0), 3 (T3), 6 (T6) months, and around the delivery for mothers. Urine samples were analyzed for a total of 7 major phthalate metabolites of 5 of the most commonly used phthalates. At six months of age a global child development assessment using the third edition of the Griffith Scales of Child Development (GSCD III) was performed in 104 participants.

**Results:**

In a total of 387 urine samples, the seven metabolites analyzed appeared widespread and were detected in most of the urine samples collected at any time of sampling (66-100%). At six months most of the Developmental Quotients (DQs) falls in average range, except for the subscale B, which presents a DQ median score of 87 (85-95). Adjusted linear regressions between DQs and urinary phthalate metabolite concentrations in mothers at T0 and in infants at T0, T3 and T6 identified several negative associations both for infants’ and mothers especially for DEHP and MBzP. Moreover, once stratified by children’s sex, negative associations were found in boys while positive in girls.

**Conclusions:**

Phthalates exposure is widespread, especially for not regulated compounds. Urinary phthalate metabolites were found to be associated to GSCD III scores, showing inverse association with higher phthalate levels related to lower development scores. Our data suggested differences related to the child’s sex.

## 1 Introduction

Phthalates are synthetic chemical compounds obtained through a process of esterification between phthalic anhydride and an alcohol of variable size, from methanol to tridecanol. This family of non-persistent chemicals are generally used as plasticizers to give flexibility, malleability and temperature tolerance to several materials, such as polyvinylchloride (PVC) (1).

Phthalates have a weak interaction with the polymer matrix where they are dispersed and so they are quickly released from their plastic embodiment into the environment. Since they are used in a wide range of everyday consumer products, these compounds are considered ubiquitous pollutants. Consequently, the potential human exposure occurs through multiple sources and is widespread, as well as continuous over time (2–4). One of the main sources of exposure during neonatal life is represented by breast or formula milk. During weaning, exposure due to food contamination is also frequent. Moreover, in the first months and years of life, ingestion from non-food origin occurs due to some typical newborn and toddler behaviors (use of pacifiers and teethers, frequent hand-to-mouth contact, crawling on vinyl and household floors) (5, 6). Phthalates are easily absorbed, distributed by circulation and quickly metabolized by intestinal lipase and esterases into their respective primary and secondary metabolites, and finally eliminated, mainly in the urine (7, 8).

Phthalates are classified as Endocrine-disrupting Chemicals (EDCs) because they may interfere with the regular hormonal activity. In particular, phthalates compete with endogenous hormones-receptor binding and with hormone transport proteins modifying normal gene expression within the cell (9–13). For this reason, over the past decades, European and international regulations have worked on restricting their use in products such as toys, childcare products, food packaging materials and so on (14).

Early life exposure to EDCs, and in particular to phthalates, is suspected to affect child health including neurodevelopment (15–19). The risk is higher when exposure occurs during windows of vulnerability, such as prenatal, perinatal, and early childhood life. In fact, during the third trimester of pregnancy the brain growth spurt (BGS) occurs. Moreover, the development of the central nervous system (CNS) is completed within the first two years after birth. During this time, the CNS is particularly susceptible to external stressors, which if present, can influence neurodevelopment in different domains (i.e. sensory, motor and cognitive functions) (20–24). Several epidemiological studies have described harmful associations between prenatal exposure to phthalates and child behavior or cognition (25, 26).

The aim of this study is to analyze the relationship between the urinary levels of phthalate metabolites and the global development measured by the Griffiths Scales of Children Development (GSCD) at six months.

## 2 Methods

### 2.1 Participants and sample collection

Participants were recruited as part of the Modena cohort study, a longitudinal cohort study started in 2019 with the aim of investigating the relationship between peri and postnatal exposure to phthalates and the possible relationship with hormonal, anthropometric and neurocognitive development, in healthy Italian children during the first 3 years of life. Mothers were recruited during their hospital stay in the Obstetric ward between March 2019 and October 2020, as soon as possible after delivery. Newborns with mothers of legal age (>18 years old) at delivery, who understood the Italian language and had a singleton pregnancy were eligible for participation in the main cohort study. Other inclusion criteria were: at term delivery (37-41 weeks), appropriate-for-gestational-age (AGA) infant, Apgar score >7 five minutes after birth.

The study was approved by Area Vasta Emilia Nord Ethics Committee (2018/num715). Informed consent to participation and publication of the data was obtained from caregivers of all individual participants included in the study.

The study is a sub-sample of the Modena cohort study. The eligibility criteria were: 1. having at least one urine sample for exposure assessment from birth to 6 months or from the mother within a few days from delivery and 2. having completed the global development assessment with GSCD at six months. Sample size calculation was performed for the main study, and it was estimated that about 200 newborns were needed. Therefore, we enrolled 197 mothers and child pairs. Among them, 104 infants completed the global development assessment at 6 months, and this determined the sample size for the present sub-study.

### 2.2 Urine sampling for phthalate exposure assessment

Children underwent consecutive examinations, from birth to sixth month of age, at respectively 0 (T0), 3 (T3), 6 (T6) months. At each evaluation spot urine samples were collected.

Urine samples were chosen as preferred biological matrix to assess phthalate metabolites concentration in mothers and children (27). Children spot urine samples were collected, using phthalate-free pediatric urine bags attached to the skin of external genitalia. Mothers were asked to provide a spot urine sample in phthalate-free plastic containers within a few days from delivery. Urine samples obtained by newborns were collected by trained hospital personnel during their hospital stay, as well as during follow-up visits, until the COVID pandemic outbreak. From that moment on, parents were instructed on correct at-home sampling techniques and were provided with tested phthalate-free urine sampling kits. Once the sample was obtained, it was collected by researchers and immediately labeled and stored in glass vials at -20°C until extraction and analysis procedures.

### 2.3 Phthalate analysis

Urine samples were analyzed for a total of 7 major phthalate metabolites of 5 of the most commonly used phthalates (**Table 1**). Urine sample preparation was performed in the laboratories of the Department of Biomedical, Metabolic and Neural Sciences, while phthalate qualitative and quantitative determination was conducted at the CIGS (Centro Interdipartimentale Grandi Strumenti) of the University of Modena and Reggio Emilia. Abbreviation on phthalates, metabolites and their characteristics may be found in **Table 1**. Analytical procedures for sample preparation were optimized following published methods by the USA Centers for Disease Control and Prevention (CDC)_Environmental Health Department. Urine samples were defrosted at room temperature sampling 1 ml of urine. β-glucuronidase enzyme was used to hydrolyze the glucuronidated phthalate metabolites (adding 200 μL of 6% β-glucuronidase enzyme in 6.5 pH acetate buffer and then using 10 ppb of internal standard). They were incubated for 120 minutes at 38°C to allow the cleavage of monoester glucuronides by the enzyme. The reaction was blocked by adding glacial acetic acid and 5% acetonitrile in ultrapure water to the sample solution. The samples were then purified by solid phase extraction (SPE), in order to prevent impurities that may affect matrix compounds and alter the sample selectivity and specificity. The cartridges were conditioned with 2 mL of methanol and 2 mL ultra-pure water. Urine sample was then loaded into the cartridge phase which aims to block interferents and phthalates. The cartridge was then washed with 2 mL of 0.1% acetic acid in ultra-pure water. The samples were eluted with 1 mL of 0.1% acetic acid in acetonitrile. All phthalate metabolites were quantified using reversed-phase-high-performance liquid chromatography–mass spectrometry (RP-HPLC–MS).

**Table 1:** Names, abbreviations, molar weight and limit of detection (LOD) of investigated phthalate parents and their metabolites.

Phthalate diester	Urine metabolites	Abbreviation	Molar weight (g/mol)	LOD (ppb)
Di-methyl phthalate		DMP	194.18	
	Mono-methyl phthalate	MMP	184.18	0.1
Di-ethyl phthalate		DEP	224.24	
	Mono-ethyl phthalate	MEP	194.18	0.1
Di-n-butyl phthalate		DnBP	278.34	
	Mono-n-butyl phthalate	MnBP	222.24	0.05
Butylbenzyl phthalate		BBzP	312.36	
	Mono-benzyl phthalate	MBzP	256.257	0.05
Di-(2-ethyl-hexyl) phthalate		DEHP	390.56	
	Mono-(2-ethyl-hexyl) phthalate	MEHP	278.34	0.25
	Mono-(2-ethyl-hydrohexyl) phthalate	MEOHP	292.33	0.05
	Mono-(2-ethyl-oxohexyl) phthalate	MEHHP	294.34	0.05

Ppb, parts per billion.

### 2.4 Assessment of neurocognitive development

From 6 months onwards, the study design included a global child development assessment of the enrolled children using the third edition of the Griffith Scales of Child Development (GSCD III) (28, 29). This widely used specialistic tool explores five key evolutionary areas: foundations of learning (scale A), language and communication (scale B), eye and hand coordination (scale C), personal-social-emotional (scale D), and gross motor (scale E). Subscale scores and a total score, the so called General Development Score (GDS), were calculated. Subscale and GDS raw scores were standardized for sex and age according to GSCD normative scoring tables and guidelines, and standardized “Developmental Quotient (DQ)” scores were produced. In the present study, we used the Italian validated version GSCD questionnaire addressed to 0–12 months children and the normative scoring tables produced for the Italian population (30, 31).

According to the Griffiths III administration manual, the observed standardized DQ can be divided into seven categories for score interpretation: Extremely High (DQ ≥ 130), High (DQ = 120-129), Above Average (DQ = 110-119), Average (DQ = 90-109), Below Average (DQ 80-89), Borderline (DQ = 70-79), Extremely Low (DQ ≤ 69). In the present study, a trained team of pediatricians and a psychologist administered the GSCD at the six-month follow-up visit. After February 2020 due to pandemic restrictions, some of the 6-month follow-up visits were performed online. Staff and families were trained to accurately reproduce the procedures and assessments of in-person GSCD visits.

### 2.5 Covariates

Given that child development is a complex process due to the interaction with multiple factors derived from the environment, society and the family background, we collected as covariates the items that majorly interact with the GSCD paradigm of development, and which are related to social-economical-cultural background of the parents (31). Data on socio-demographic characteristics of caregivers was collected during the enrollment visit. To specifically examine the relationship between the parents’ cultural background and the baby’s cognitive performance according to the GSCD scales, we defined three educational levels (1 = up to middle school; 2 = high school; 3 = bachelor or more). Working groups were defined according to European Socio-economic Groups (ESeG) (32) classification composed of 3 classes of 9 categories: high class (managers, professionals), middle class(technicians and associated professionals employees, small entrepreneur), working class (clerks and skilled service employees, skilled industrial employees, lower status employees) and not working. Maternal age at delivery, pre-gravidic weight, weight at delivery and height were collected for each mother and pre-gravidic BMI was calculated. Lifestyle habits of mothers during pregnancy were collected. Finally data on whether the evaluation was conducted before or after the pandemic outbreak was collected. Variable details are described in **Table 2**.

**Table 2:** Demographic-socio-econimic characteristics of infants and caregivers.

		Overall N = 104*^1^*
Child characteristics		
Child sex	female	43 (41%)
	male	61 (59%)
Gestational age at delivery	(weeks)	39 (39, 40)
	Not specified	1
Delivery mode	Caesarean	23 (22%)
	Natural	81 (78%)
Weight Percentile at birth*		57 (35, 72)
Height Percentile at birth*		60 (47, 74)
Head Circumference Percentile at birth*		83 (54, 83)
Maternal characteristics		
Mother’s age at delivery	(years)	34 (31, 37)
Mother Citizenship	Italian	98 (94%)
	Not Italian	6 (5.8%)
Mother’s Educational level	Up to Middle School	1 (1.0%)
	High School	29 (28%)
	Bachelor or more	74 (71%)
Mother’s Working status**	High class	35 (34%)
	Middle class	19 (18%)
	Not working	13 (12%)
	Working class	37 (36%)
Mother’s BMI before pregnancy	<18.5	4 (3.9%)
	18.5-<25	77 (75%)
	25-<30	16 (16%)
	>=30	6 (5.8%)
	Not specified	1
Smoking during pregnancy	No	98 (95%)
	Yes	5 (4.9%)
	Not specified	1
Alcohol consumption during pregnancy	No	80 (77%)
	Yes	24 (23%)
Paternal characteristics		
Father’s age	(years)	37 (34, 41)
Father Citizenship	Italian	100 (96%)
	Not Italian	4 (3.8%)
Father’s Educational level	Up to Middle School	4 (4.2%)
	High School	49 (51%)
	Bachelor or more	43 (45%)
	Not specified	8
Father’s Working status**	High class	25 (27%)
	Middle class	11 (12%)
	Not working	1 (1.1%)
	Working class	56 (60%)
	Not specified	11

n(%) or median (IQR).*Antropometric percentiles according to WHO (33) **According to the European Socio-economic Groups (ESeG) classification (32). ^1^number of participants.

### 2.6 Study size

The Present study involves 104 participants.

### 2.7 Statistical analysis

Categorical variables characteristics were summarized by absolute and relative frequencies. Median and interquartile range (IQR) were used to summarize continuous variables. Phthalate concentrations below the respective level of detection (LOD) are expressed as the LOD value divided by the square root of 2 (<LOD= LOD/√2). LOD values for each metabolite are reported in **Table 1**. Metabolites of DEHP were expressed combined as the sums ∑DEHPm, by using equation (1):


$$\sum DEHP=\left[\left(\frac{UC_{m1}\left(\frac{ng}{mL}\right)}{MW_{m1}\left(\frac{ng}{nmol}\right)}\right)+\left(\frac{UC_{m2}\left(\frac{ng}{mL}\right)}{MW_{m2}\left(\frac{ng}{nmol}\right)}\right)+\cdots \right]\times MW_{p}(\frac{ng}{nmol})$$

where ∑DEHPm is the summed urinary concentration of metabolites of DEHP (MEHP, MEHHP, MEOHP) expressed as the urinary concentration of parent compound; UCm1, UCm2 etc. are the urinary concentration of the individual metabolites and MWm1, MWm2 etc. indicate molecular masses of each metabolite, respectively, while MWp is the molecular mass of the parent compound. Molecular masses are reported in **Table 1**.

When considering phthalate metabolite concentration, aside from descriptive analyses, statistical analyses were conducted after natural logarithmic transformation to minimize the influence of highly skewed data. Pearson’s chi-square test or Fisher’s exact test was used to compare categorical variables. T-student test or Wilcoxon rank sum test (also known as Mann Whitney U test) were used to analyze differences in two groups of numeric variables depending on their distribution. We examined potential confounding variables by literature search and included those factors likely to be associated both with phthalate exposure and adverse neurodevelopment (details in **Table 2**). Given the relatively small sample size, we ran regression models with all candidate covariates and no exposure with each GSCD score. Covariates with p-values less than 0.20 in at least one of the models were retained in all final models, The retained covariates were: child sex, gestational age at delivery, delivery mode, mother’s educational level, father’s educational leveland if GSCD was performed before or after the pandemic outbreak.

Several studies reported effect modification for numerous neurodevelopmental outcomes depending on the sex of the child (34–40), We performed regression models stratifying population by sex.

Finally, some of the visits (n:35) were conducted online due to pandemic restrictions. Staff attempted to replicate the procedures and assessments of in-person GSCD visits as accurately as possible; however, given the possibility of bias in test evaluation during online visits, as well as the higher variability of the scores producing some outlier scores, we performed a subgroup analysis on infants visited exclusively in presence. This affected our sample size, but it allowed us to exclude outliers from the analysis. False discovery rate was controlled by using the Benjamini-Hochberg procedure.

Missing data were dealt with pairwise-case data analysis. Statistical software IBM SPSS Statistics^®^, version 27 and the R software version 4.2.1 (The R Foundation for Statistical Computing) for plotting and advanced statistical analysis, with packages including tidyverse, ggplot2, ggh4x, gtsummary. Code for linear model figure plotting was adapted from Rolland et al. (41).

## 3 Results

### 3.1 Description of the population

**Table 2** describes the overall population of our study involving 104 children, with regards to demographic-socio-economic characteristics of infants and caregivers.

The comparison between the 104 children who underwent the GSCD III evaluation at T6 and were included in the present study (the *‘included’* group), and those who were not is described in **Supplementary Table S1**. Significant differences between the two groups were detected for mother’s scholarization (higher educational level in the included group), pre-gravidic BMI (higher normo-BMI in the included group) and smoking habit during pregnancy (lower smoking in the included group). No differences were found for the paternal socio-economic characteristics.

### 3.2 Urinary phthalate metabolite concentrations

For the present study we collected 387 urine samples from 103 mothers, 95 newborns and respectively 92 and 97 infants of three and six months.

Metabolite concentrations detected in urine samples of mothers and children overtime are presented in **Table 3**. In a total of 387 urine samples, the seven metabolites analyzed appeared widespread and were detected in most of the urine samples collected at any time of sampling (66-100%).

**Table 3:** Phthalate metabolite concentration (ng/mL) in 387 urine samples from mothers close to delivery, and in their children at birth, three and six months.

	% > LOD	Median (IQR)
Mother samples at delivery (M) - n: 103		
**Sampling time from delivery (days)**		1.00 (0.00, 1.00)
**MMP**	66	0.22 (0.07, 0.42)
**MEP**	100	15 (5, 30)
**MnBP**	92	5 (2, 10)
**MBzP**	99	2.46 (1.13, 5.56)
**∑DEHP**	100	4 (2, 8)
Newborn samples at birth (T0) - n: 95		
**Sampling time from birth (days)**		1.00 (0.00, 1.00)
**MMP**	67	0.18 (0.07, 0.34)
**MEP**	100	15 (9, 31)
**MnBP**	92	7 (3, 14)
**MBzP**	99	4 (1, 8)
**∑DEHP**	97	3.0 (1.6, 5.2)
Infant samples at 3 months (T3) - n: 92		
**Sampling time from birth (days)**		96 (90, 103)
**MMP**	87	0.19 (0.13, 0.42)
**MEP**	100	14 (8, 36)
**MnBP**	100	2.5 (1.5, 4.5)
**MBzP**	95	0.64 (0.25, 1.56)
**∑DEHP**	100	2.2 (1.2, 4.8)
Infant samples at 6 months (T6) - n: 97		
**Sampling time from birth (days)**		196 (189, 210)
**MMP**	96	0.40 (0.21, 0.76)
**MEP**	100	19 (8, 57)
**MnBP**	100	3.9 (2.2, 6.9)
**MBzP**	98	1.2 (0.6, 2.8)
**∑DEHP**	100	5 (3, 12)

Phthalate abbreviations are explained in **Table 1**.

Comparison between ‘*included*’ and *‘not included’* groups are shown in **Supplementary Table S2**. Overall, the characteristics and urinary concentrations of the *‘included’* group, did not differ significantly from the *‘not included’*.

### 3.3 Assessment of child development

Developmental quotients at 6 months, expressed as median and IQRs, are shown in **Table 4**. Due to the pandemic restrictions 35 evaluations (33.7%) were performed online, while the ‘in-person’ evaluations were 69 (66.3%). Less than 3% of the study participants had missing outcome data. According to **Table 2** most of the DQs fall in average range, except for the subscale B, which present a DQ median score of 87 (85–95). In particular, 46 infants (44%) scored in average or above, while 46 (44%) below average, 8 (7.7%) borderline and 4 (3.8%) extremely low. **Table 4** shows the standardized scores obtained at 6 months for the overall population and categorized by children’s sex. The General development and the scales A and D scores, are significantly higher in males; while the scale C had significantly higher score for females. For scale D there is a median score of 93 in boys, which is in average, while in girls there is a median score of 86, which falls below average.

**Table 4:** Griffiths III Scales of Child Development results stratified by child sex.

	Overall, N=104^1^	Female, N=43^1^	Male, N= 61^1^	p-value^2^
General Development Score *	97 (92, 100)	95 (90, 97)	98 (93, 100)	0.028
**Scale A: Foundation of Learning**	105 (101, 114)	101 (94, 106)	111 (105, 114)	<0.001
**Scale B: Language and Communication**	87 (85, 95)	87 (81, 88)	91 (85, 97)	0.2
Scale C: Eye and Hand Coordination*	101 (95, 106)	106 (98, 106)	101 (95, 103)	0.005
Scale D: Personal-Social-Emotional*	90 (85, 97)	86 (81, 90)	93 (87, 99)	<0.001
Scale E: Gross Motor*	100 (95, 109)	101 (95, 109)	100 (96, 106)	0.6

^1^Median (IQR); ^2^ Wilcoxon rank sum test. *Missing data for Griffiths III Scales of Child Development: 3 for General Development Score (GDS) given by 2 missing data in subscale D and E, and 1 in subscale C

### 3.4 Adjusted associations between urinary phthalate metabolite concentrations and Griffith’s developmental quotients scores

**Figure 1:**
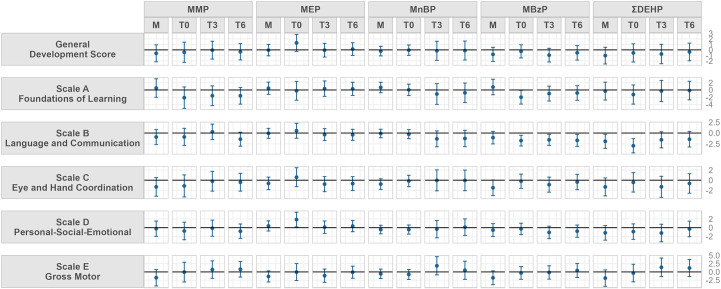
Model adjusted for child sex, gestational age at delivery, delivery mode, mother’s educational level, father’s educational level and if GSCD was performed before or after the pandemic outbreak. Beta and 95% confidence interval of adjusted linear regression models between urinary concentrations of phthalate metabolite and parent compound and Griffith Scales of Child Development (GSCD) measured at 6 months in the Modena cohort study, stratified by sampling timing (from mothers close to delivery, M, and in their children at birth, T0, three, T3, and six months, T6). Values may be found in **Supplementary Material; Table S4**. Phthalate abbreviations are explained in **Table 1**.

**Figure 1** shows the adjusted linear regressions between Griffith’s developmental quotients scores and urinary phthalate metabolite concentrations in mothers at T0 and in infants at T0, T3 and T6. Several trends both for infants’ and mothers concentrations were found, for each exposure period. Numerical betas and confidence intervals are shown in **Supplementary Table 4**.

Maternal urinary MnBP concentrations showed a negative trend with the scale C scores at six months while MBzP concentrations showed a negative trend with scale B, scale C and scale E scores. Maternal DEHP concentrations were negatively associated to scale B scores at six months.

At T0, neonatal concentrations of MBzP and DEHP metabolites showed an inverse trend to the scale B at T6. MBzP also showed negative trends with scale A scores. Neonatal MEP concentrations at T0 were positively associated to scale D scores.

MnBP urinary concentrations at T3 showed positive trends with scale E at three and six months; MBzP for all sampling timings was negatively associated to subscale B scores.

**Figure 2:**
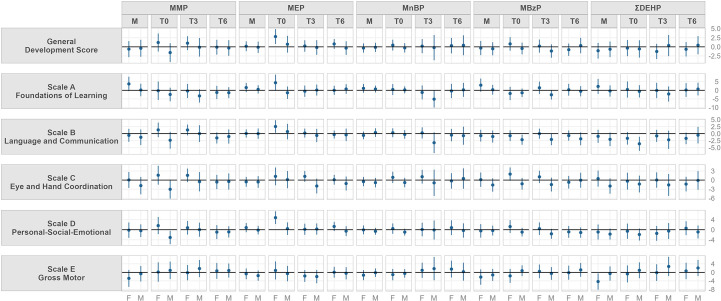
Model adjusted for gestational age at delivery, delivery mode, mother’s educational level, father’s educational leveland if GSCD was performed before or after the pandemic outbreak. Beta and 95% confidence interval of adjusted linear regression models stratified by sex between urinary concentrations of phthalate metabolite and parent compound and Griffith Scales of Child Development (GSCD) measured at 6 months in the Modena cohort study, stratified by sampling timing (from mothers close to delivery, M, and in their children at birth, T0, three, T3, and six months, T6). Values may be found in **Supplementary Material; Table S5, 6**. Phthalate abbreviations are explained in **Table 1**.

**Figure 2** shows the adjusted linear regressions between Griffith’s developmental quotients scores and urinary phthalate metabolite concentrations in mothers at T0 and in infants at T0, T3 and T6 stratified by children’s sex. Numerical betas and confidence intervals are shown in **Supplementary Table 5** for female and in **Supplementary Table 6** for male.

Maternal urinary concentrations had a positive relationship with female scale A scores for MMP and MBzP, while showed a negative association to scale E for DEHP. In boys, negative trends were found for scale C and maternal MMP, MBzP and DEHP concentrations, and for scale B and D and DEHP concentrations.

Neonatal urinary concentrations (T0) showed a positive trend in females with: scale C for MMP, General development score, scale A, B, C and D for MEP, scale C for MBzP. Moreover, in girls a negative trend was found between scale E and MBzP.

In males, neonatal urinary concentrations (T0) were found to be negatively associated to: scale B, C and D for MMP; scale B for MBzP and for DEHP.

At three months of age (T3) a positive trend between female urinary MMP concentrations and scale C was found.

In males, negative associations at T3 was found between MnBP and MBzP and scale A and B.

Urinary phthalate concentrations at T6 did not show relevant trends with DQs at six months in girls and boys.

Finally, given the possible bias in test evaluation during online visits, we performed a subgroup analysis on infants visited exclusively in presence. Even if due to sample size reduction relationships appear less strong, overall associations and patterns were similar to those found in the main models.

## 4 Discussion

### 4.1 Main findings

The present study evaluates peri- and postnatal exposure to phthalates in an Italian pediatric cohort of full term babies and their mothers and its possible associations with the child’s neurodevelopment. The population showed homogeneous characteristics: all the babies were born from a single center, at term, with no perinatal complications. There is a relatively equal division between males and females and the anthropometric characteristics were in line with healthy newborns for both sexes. As shown in **Supplementary Material**, few differences between ‘*included*’ and ‘*not included*’ groups were found, in particular for mothers (educational status, pre-gravidic BMI and smoking habits). Despite these differences, phthalate metabolites concentrations resulted almost equally distributed within the two groups (as shown in **Supplementary Table S2**). Our results showed that infants and mothers were exposed to several phthalates during the whole study period.

#### 4.1.1 Neurocognitive development

The neurocognitive assessment performed for 104 children at six months through the Griffith Scales of Child Development (GSCD III) showed scores within the normal range for General development score and scales A, C, D, and E. This was expected because the children involved in our study had no significant neurodevelopment risk factors, due to the study inclusion criteria.

For scale B, 46% of our population scored below average. This result could therefore be justified due to the social distancing measures introduced by the pandemic outbreak and the consequent prolonged reduced socialization (42–44). In addition, the use of the facial mask may have influenced and slowed learning, since it hinders labial vision. The evidence that COVID-19 somehow affected children’s neurocognitive development was shown in a recent cross-sectional study. In fact, significantly lower scores in both general development and subscales scores were observed in children assessed during the pandemic period (31).

The surrounding environment, external cues, and lifestyle habits are very important in determining and eliciting neurocognitive development in children. In a study conducted on Filipino children, the Griffith’s Mental Developmental Scales were evaluated by comparing the performance of 742 Filipino children longitudinally at 6, 12 and 24 months old to those of their British counterparts.

Although the performance of the Filipino children in the Griffiths test were within average for age, their performances on developmental sub quotients at later ages of 12 and 24 months were significantly lower than British children and may have been influenced by differences in ethnicity, cultural traditions and limited environmental resources (44).

Our results have also shown differences in the GSCD III scores according to sex, although scores are adjusted for age and sex.

In particular, males reported higher scores in scale A (Foundations of Learning) in scale D (Personal-Social-Emotional) and in General developmental score; while girls, reported higher scores in scale C (hand-eye coordination). From an overall view, in our population boys have higher scores than girls in different areas of development. It is known that there are anatomical and functional differences between the central nervous system maturation of males and females. Hormonally dependent, sex-specific changes in the ultrastructure of the developing central nervous system are known. Higher prenatal androgens in males than in females modulates the masculinization of external genitalia, the developing neural system, and the behavioral development. In particular, the androgen levels during critical periods in early development are thought to contribute to the between- and within-sex variability in human social and cognitive behavior (4, 45). In literature, data comparing the differences between neurodevelopmental assessment of male and female are present, especially using a different tool compared to our study, the Bayley III scales (40). From a general overview, females present higher scores for language and fine motor performance, while males for gross motor functions and oculo-manual coordination, although these differences are quite clear from 12 months onwards (46).

In a 2019 Danish study (45), the authors investigated whether gender differences were present in Bayley-III scores and behavior in a sample of typically developing children at ages 4, 7, 10, 13, 24 and 36 months. The results demonstrated the existence of gender differences in test scores, but the pattern of differences varied across scales and subtests. In the cognitive scale, gender differences were found at 24 and 36 months with higher scores in girls rather than boys; in the language scale at 10, 13, 24 and 36 months, which found girls’ performances to be superior to those of the boys as well as in the motor scale at 36 months. In a Chinese study, conducted in 2019 to detect possible sex differences in term children using the Bayley-III cognitive scales, 1486 children aged 16 days to 42 months were analyzed. In this study, it was found that girls in the 0-5, 6-11, 12-17 and 18-23-months ranges reported higher scores than boys, although the only significance was reported in the 18-23 months range (38). It is therefore necessary to investigate further, particularly with regard to the use of the GSCD III in the assessment of healthy, term-born children.

#### 4.1.2 Possible associations between phthalates exposure and neurocognitive development

This study detected several possible associations between urinary phthalate concentrations in mothers and children, at three different time points, and GSCD III scores conducted at 6 months.

A large number of tests were carried out, hence, we cannot exclude that some statistically significant results were random. Furthermore, the small sample size may account for the change in significance observed between the unadjusted and adjusted model. For this reason, attention should be focused mainly on the more consistent associations.

As shown in **Figure 1**, MBzP and DEHP metabolites seem to be the most negatively associated to GSCD III, in particular to scale B and C. MBzP itself also showed a negative association with scale A and E. As previously mentioned, these are two of the most regulated phthalates due to their anti-androgenic and endocrine disrupting effects. Although found at low concentrations, both MBzP and DEHP metabolites have shown significant negative associations with different scales of development, confirming previous literature data (47).

MMP, MnBP, DEHP showed positive relation with scale E. The positive association to the gross motor function may be explained with the tendency at six months, for good scale-E performants, to bring objects to the mouth, to suck toys and to start exploring the playground. Maternal levels of MnBP were also found to be negatively associated to scale C, enforcing the negative effect of MBzP and DEHP on neurodevelopment (48).

MEP at T0 was found to be positively associated to scale D performed at six months. MEP is a metabolite of one of the not restricted phthalate (DEP) currently used mainly in self-care products. Our personal interpretation of this direct association with scale D, is linked to the high level of personal cure and hygiene that families of babies with high scores at scale D may present. The more the baby is keen to socialization, the more probably the parent are keen to cure themselves and the baby and vice-versa. However no results in literature are available to confirm this possibility (49). Child development is an ongoing process within the first years of life, influenced by several different factors as: the relationship with parents and care-givers, appropriate sensorial stimulations or unappropriated deprivation, energy intake, quality of sleep etc. Being able to detect an association within phthalates exposure and GSCD III scores at six months of age in otherwise healthy children with no apparent neuro-developmental risk factors, brings us to be very careful in results interpretation. However, as clinicians, we need to consider these findings as an alert for the future child development. We need to ask ourselves if GSCD III lower scores are due to a delayed acquisition of the development milestones or to a proper modulation of the neurodevelopment process. Moreover, seen that phthalates are ubiquitous, our children will experienced a prolonged exposure to chemical compounds during a very vulnerable temporal window, leading to a possible summatory effect. Longitudinal follow up of children becomes a priority for brain health.

Once we stratified the population according to sex, associations between GSCD III scores and phthalates concentrations changed. In particular, from an overall view of **Figure 2** we detected positive associations in female, while negative associations in male.

MMP, MnBP, MBzP and DEHP metabolites showed several negative associations with all the scale of GSCD III in male, during all the different timing of the study.

These negative associations were previously detected in literature, indicating that there is a negative influence of these compounds on neurodevelopment, especially in males, probably linked to the anti-androgenic effects of many of them.

A recent study suggested that prenatal exposure to phthalates could be inversely associated with the MDI and PDI of infants, particularly in males, at 6 months (34). Other studies (35–40) showed a negative correlation between phthalate levels and neurodevelopment: in all cases a significant inverse associations between prenatal exposure to DBP and cognitive and psychomotor development was found.

On the contrary, positive associations between GSCD III scores in females and urinary phthalates concentrations were found. This was seen especially for MEP, who showed associations with general development score, scale A, B and D for neonatal urinary concentrations.

In females, negative association were found with scale E and MBzP and DEHP concentrations.

Similar associations were previously found by Hyland C et al, who found slightly higher IQ scores in girls with higher phthalates concentrations (50) compared to boys, although age of assessment and urinary collection was completely different from our study. In fact, we need to remember how, in our population, general quotient, scale A and scale D scores were higher in male.

The positive associations in girls may depend on the different body composition of female neonates and children, who present an higher percentage of body fat mass, than male peers.

Differences in body composition between female and male neonates are confirmed, with girls having a higher fat mass divided by total body mass (%) (11.1% vs. 9.6%) and lower free fat mass (2827 g vs. 2979 g), which seems to be maintained throughout life (51, 52).

Full term males are heavier at birth and have a higher lean body mass, whereas females have more subcutaneous fat (53). These sex-related differences have been primarily attributed to the action of fetal sex steroid hormones, which presumably enhances lean body mass growth *in utero* and later during the first months of life, determining a metabolic imprinting of the body fat mass in males (54).

Boys may be more vulnerable to the neurotoxicity in several areas of development, due to anti-androgenic effects of phthalates, although their global scores were within the normal range except for scale B (55–57).

Females may be keener to the accumulation of phthalates in body fat mass. This may lead to a positive association within phthalates concentrations and different developmental domains, although lower scores than males were often found, except for scale C.

### 4.2 Strengths and limitation

The small number of subjects enrolled in the study is strictly dependant by the complexity of urine sampling in newborn during their very first days of life, which is especially true when conducting stratification by sex. Given this problem, even if the sample appears small, this study is one of the few European studies to assess exposure at this timepoint and, at the moment, one of the largest (34–39). COVID-19 disruption of study protocol affected timing and logistic of sampling collection. After the outbreak, caregivers were asked to collect urine samples. To avoid contamination, we carefully trained them on proper sample techniques and provided them with phthalate-free sampling kits. Finally, researchers collected and stored urine samples as quickly as feasible after sampling. While attempts were made to minimize contamination in our investigation, the chance of contamination exists. Secondly, the use of single spot urine, especially with short half-life compounds, limits the generalizability of our findings to everyday life or chronic exposure. The use of multiple single spot urine over the first six months of life enabled us to assess time trends in early life exposure to phthalates. Furthermore, some authors argued that a single sample may also reasonably predict average metabolite concentrations over a period of several months (58–60). Even though the first year of life is a critical period for brain development, very few studies assessed phthalate concentrations during the first year of life (41, 61), hence the collection of three sample points in the first six months of life is a great strength of our study.

It is noteworthy that the associations of urinary phthalate metabolites collected at 6 months and the GSCD scores collected at the same timing are cross-sectional and need to be interpreted with caution.

Due to the presence of many exposures as well as outcomes, multiple comparisons were performed and we cannot exclude that the some of the association we found were due to chance. Associations found with MMP must be interpreted cautiously, given that the percent of detected samples but lower than LOD was lower than 90%.

Finally, the collection of information regarding the family’s socio-economical-cultural background, perinatal and child factors, allowed us to adjust for potential confounder. However, given the complexity of environmental exposure, there may be confounding from residual covariates.

## 5 Conclusion

Our results highlighted how phthalates exposure in the restricted geographic area analyzed, is widespread. At six months of age, our population of healthy term newborn scored within the normal range in all the different domains of development, except for the language development. This could be related to the social distancing and the use of masks due to pandemic restrictions.

Urinary phthalate metabolites were found to be associated to GSCD III scores, showing negative associations within ‘language’ and ‘hand-eye coordination’ and MBzP and DEHP concentrations. The impact of phthalates on neurodevelopment showed a clear sex-related difference with several negative associations in boys within GSCD III scores and anti-androgenic phthalates, while several positive associations in girls, especially with non-regulated compounds.

As phthalates are almost ubiquitous compounds and it is therefore quite impossible to avoid them in daily life, some precautions can be used to minimize exposure. Intervention strategies to reduce the exposure to phthalates in children may include: to prefer fresh foods, to avoid the use of environmental deodorants or cosmetics and personal care products if not phthalates-free; to pay attention to the labels of products purchased, making sure they are phthalate-free; to dispose of older toys or food contact materials that were made prior to the introduction to phthalate regulations, preferring the use of materials that come from countries that currently have in place regulations that limit the use of such compounds (I.e.: European Union)t (62).

## Data availability statement

The raw data supporting the conclusions of this article will be made available by the authors, without undue reservation.

## Ethics statement

The studies involving human participants were reviewed and approved by Comitato Etico Area Vasta Emilia Nord. Written informed consent to participate in this study was provided by the participants’ legal guardian/next of kin.

## Author contributions 

LL, LP, ER conceptualized and designed the study, coordinated and supervised data collection, drafted the initial manuscript, and reviewed and revised the manuscript. EP, VT, FC, EF, GT and NB collected data. AF analyzed urinary samples. LL, LP and ER critically analyzed and interpreted data. FF, BP and LI critically reviewed the manuscript. All authors approved the final manuscript as submitted and agree to be accountable for all aspects of the work. LL and LP have contributed equally to this work and share first authorship. All authors contributed to the article and approved the submitted version.
