# Supplementary material for: Perinatal and postnatal exposure to phthalates and early neurodevelopment at 6 months in healthy infants born at term
Source: Front Endocrinol (Lausanne). 2023 May 24;14:1172743. doi: 10.3389/fendo.2023.1172743 (PMC10244530; doi:10.3389/fendo.2023.1172743)
Supplement: Supplementary file 1 [file Table_1.docx]

Supplementary Material

**Title: Perinatal and postnatal exposure to phthalates and early neurodevelopment at 6 months in healthy infants born at term.**

**Authors**: Lucaccioni L^1§^, Palandri L^2,3§^, Passini E^4^, Trevisani V^4^, Calandra Buonaura F^5^, Bertoncelli N ^6^, Talucci G^6^, Ferrari A^2^, Ferrari E^2^, Predieri B ^1^, Facchinetti F^7^, Iughetti L^1,4^*****, Righi E^2^.

§ Equal contribution and first authorship

***Corresponding Author**: Prof. Lorenzo Iughetti, Department of Medical and Surgical Sciences of the Mother, Children and Adults. University of Modena and Reggio Emilia. Modena, Italy. [lorenzo.iughetti@unimore.it](mailto:lorenzo.iughetti@unimore.it)

# Supplementary Tables

Table S1: Study population descriptive table, with comparison between the Modena Cohort families whose child were included in the present study and those that were not.
Inclusion criteria included: Having at least one sample at one sampling point and having performed Griffiths Scales of Child Development (GSCD) evaluation at 6 months.

|  |  | **Included**  **N = 104*^1^*** | **Not Included**  **N = 84^1^** | **p-value^2^** |
| --- | --- | --- | --- | --- |
| **Child characteristics** |  |  |  |  |
| **Child sex** | female | 43 (41%) | 41 (49%) | 0.3 |
|  | male | 61 (59%) | 43 (51%) |  |
| **Gestational age at delivery** | (weeks) | 39 (39, 40) | 39 (39, 40) | 0.7 |
|  | Not specified | 1 | 8 |  |
| **Delivery mode** | Caesarean | 23 (22%) | 16 (21%) | 0.8 |
|  | Natural | 81 (78%) | 62 (79%) |  |
|  | Not specified | 0 | 6 |  |
| **Weight Percentile at birth*** |  | 57 (35, 72) | 56 (38, 72) | 0.8 |
|  | Not specified | 0 | 1 |  |
| **Height Percentile at birth*** |  | 60 (47, 74) | 68 (47, 84) | 0.6 |
|  | Not specified | 0 | 1 |  |
| **Head Circumference Percentile at birth*** |  | 83 (54, 83) | 54 (23, 83) | 0.11 |
|  | Not specified | 0 | 1 |  |
| **Maternal characteristics** |  |  |  |  |
| **Mother’s age at delivery** | (years) | 34 (31, 37) | 34 (30, 37) | 0.3 |
|  | Not specified | 0 | 2 |  |
| **Mother Citizenship** | Italian | 98 (94%) | 74 (95%) | >0.9 |
|  | Not Italian | 6 (5.8%) | 4 (5.1%) |  |
|  | Not specified | 0 | 6 |  |
| **Mother’s Educational level** | Up to Middle School | 1 (1.0%) | 12 (15%) | <0.001 |
|  | High School | 29 (28%) | 25 (32%) |  |
|  | Bachelor or more | 74 (71%) | 41 (53%) |  |
|  | Not specified | 0 | 6 |  |
| **Mother’s Working status**** | High class | 35 (34%) | 15 (19%) | 0.072 |
|  | Middle class | 19 (18%) | 25 (32%) |  |
|  | Not working | 13 (12%) | 9 (11%) |  |
|  | Working class | 37 (36%) | 30 (38%) |  |
|  | Not specified | 0 | 5 |  |
| **Mother’s BMI before pregnancy** | <18.5 | 4 (3.9%) | 11 (15%) | 0.022 |
|  | 18.5-<25 | 77 (75%) | 44 (59%) |  |
|  | 25-<30 | 16 (16%) | 11 (15%) |  |
|  | >=30 | 6 (5.8%) | 9 (12%) |  |
|  | Not specified | 1 | 9 |  |
| **Smoking during pregnancy** | No | 98 (95%) | 67 (86%) | 0.030 |
|  | Yes | 5 (4.9%) | 11 (14%) |  |
|  | Not specified | 1 | 6 |  |
| **Alcohol consumption during pregnancy** | No | 80 (77%) | 54 (69%) | 0.2 |
|  | Yes | 24 (23%) | 24 (31%) |  |
|  | Not specified |  | 6 |  |
| **Paternal characteristics** |  |  |  |  |
| **Father’s age** | (years) | 37 (34, 41) | 36.0 (34.0, 41.5) | >0.9 |
| **Father Citizenship** | Italian | 100 (96%) | 79 (94%) | 0.5 |
|  | Not Italian | 4 (3.8%) | 5 (6.0%) |  |
| **Father’s Educational level** | Up to Middle School | 4 (4.2%) | 1 (8.3%) | 0.7 |
|  | High School | 49 (51%) | 6 (50%) |  |
|  | Bachelor or more | 43 (45%) | 5 (42%) |  |
|  | Not specified | 8 | 72 |  |
| **Father’s Working status**** | High class | 25 (27%) | 1 (33%) | >0.9 |
|  | Middle class | 11 (12%) | 0 (0%) |  |
|  | Not working | 1 (1.1%) | 0 (0%) |  |
|  | Working class | 56 (60%) | 2 (67%) |  |
|  | Not specified | 11 | 81 |  |

*^1^n(%) or median (IQR). ^2^ Pearson's Chi-squared test; Wilcoxon rank sum test; Fisher's exact test.*Antropometric percentiles according to WHO (63). ** According to the European Socio-economic Groups (ESeG) classification.*

Table S2: Phthalate metabolite concentration (ng/mL) in urine samples from mothers close to delivery, and in their children at birth, three and six months. with comparison between the Modena Cohort families whose child were included in the present study and those that were not. Phthalate abbreviations are explained in Table 1 of the main article.

|  |  | **Included**  **N = 104^1^** | | **Not Included**  **N = 84^1^** | |  |
| --- | --- | --- | --- | --- | --- | --- |
|  |  | **% > LOD** | **Median (IQR)** | **% > LOD** | **Median (IQR)** | ***p^1^*** |
| **Mother samples at delivery (M)** **- n: 103** | | | |  |  |  |
| **Sampling time from delivery (days)** |  |  | 1.00 (0.00, 1.00) |  |  |  |
| **MMP** |  | 66 | 0.22 (0.07, 0.42) | 66 | 0.20 (0.07, 0.43) | >0.9 |
| **MEP** |  | 100 | 15 (5, 30) | 100 | 14 (6, 27) | 0.9 |
| **MnBP** |  | 92 | 5 (2, 10) | 96 | 5 (3, 12) | 0.3 |
| **MBzP** |  | 99 | 2.46 (1.13, 5.56) | 100 | 2.89 (1.29, 5.26) | 0.8 |
| **∑DEHP** |  | 100 | 4 (2, 8) | 99 | 4 (2, 9) | 0.6 |
| **Newborn samples at birth (T0) - n: 95** | | | |  |  |  |
| **Sampling time from birth (days)** |  |  | 1.00 (0.00, 1.00) |  |  |  |
| **MMP** |  | 67 | 0.18 (0.07, 0.34) | 65 | 0.18 (0.07, 0.31) | >0.9 |
| **MEP** |  | 100 | 15 (9, 31) | 100 | 16 (8, 40) | 0.6 |
| **MnBP** |  | 92 | 7 (3, 14) | 96 | 10 (4, 24) | 0.019 |
| **MBzP** |  | 99 | 4 (1, 8) | 100 | 4 (2, 8) | 0.7 |
| **∑DEHP** |  | 97 | 3.0 (1.6, 5.2) | 99 | 0.20 (0.12, 0.40) | 0.4 |
| **Infant samples at 3 months (T3)** **- n: 92** | | | |  |  |  |
| **Sampling time from birth (days)** |  |  | 96 (90, 103) |  |  |  |
| **MMP** |  | 87 | 0.19 (0.13, 0.42) | 87 | 0.20 (0.12, 0.40) | 0.8 |
| **MEP** |  | 100 | 14 (8, 36) | 100 | 9 (5, 16) | 0.11 |
| **MnBP** |  | 100 | 2.5 (1.5, 4.5) | 100 | 2.7 (1.5, 6.2) | 0.8 |
| **MBzP** |  | 95 | 0.64 (0.25, 1.56) | 95 | 0.58 (0.18, 0.84) | 0.3 |
| **∑DEHP** |  | 100 | 2.2 (1.2, 4.8) | 72 | 2.1 (1.3, 4.9) | 0.9 |
| **Infant samples at 6 months (T6)** **- n: 97** | | | |  |  |  |
| **Sampling time from Birth (days)** |  |  | 196 (189, 210) |  |  |  |
| **MMP** |  | 96 | 0.40 (0.21, 0.76) | 75 | 0.25 (0.11, 0.67) | 0.4 |
| **MEP** |  | 100 | 19 (8, 57) | 100 | 59 (6, 132) | >0.9 |
| **MnBP** |  | 100 | 3.9 (2.2, 6.9) | 100 | 2.0 (0.7, 6.5) | 0.3 |
| **MBzP** |  | 98 | 1.2 (0.6, 2.8) | 100 | 1.0 (0.2, 2.6) | 0.6 |
| **∑DEHP** |  | 100 | 5 (3, 12) | 100 | 4 (2, 11) | 0.6 |

^1^ Wilcoxon rank sum test

Table S3: Unadjusted linear models between urinary concentrations of phthalate metabolite and parent compound and Griffith Scales of Child Development measured at 6 months in the Modena cohort study, stratified by sampling timing (from mothers close to delivery, M, and in their children at birth, T0, three, T3, and six months, T6.

|  | **M** | | **T0** | | **T3** | | | **T6** | | | |
| --- | --- | --- | --- | --- | --- | --- | --- | --- | --- | --- | --- |
|  | β^a^ [95% CI] | p (p.adj^b^) | β^a^ [95% CI] | p (p.adj^b^) | | β^a^ [95% CI] | p (p.adj^b^) | | β^a^ [95% CI] | | p (p.adj^b^) |
| **General Development Score** | | | | | | | | | | | |
| **MMP** | -0.32 [-1.9, 1.3] | 0.704 (0.974) | -0.61 [-2.5, 1.3] | 0.521 (0.974) | | -0.27 [-2, 1.5] | 0.762 (0.974) | | -0.2 [-1.7, 1.3] | | 0.8 (0.974) |
| **MEP** | 0.092 [-0.98, 1.2] | 0.867 (0.974) | -0.071 [-1.6, 1.5] | 0.93 (0.974) | | -0.4 [-1.7, 0.93] | 0.555 (0.974) | | 0.025 [-1.1, 1.2] | | 0.966 (0.974) |
| **MnBP** | -0.55 [-1.5, 0.39] | 0.252 (0.974) | -0.77 [-1.7, 0.13] | 0.0987 (0.974) | | 0.47 [-1.2, 2.2] | 0.589 (0.974) | | 0.2 [-1.5, 1.9] | | 0.818 (0.974) |
| **MBzP** | -0.56 [-2, 0.84] | 0.432 (0.974) | 0.036 [-1.2, 1.2] | 0.954 (0.974) | | -0.0086 [-1.2, 1.2] | 0.989 (0.989) | | 0.16 [-1.1, 1.4] | | 0.803 (0.974) |
| **ΣDEHP** | -0.69 [-2.3, 0.93] | 0.404 (0.974) | -0.23 [-1.9, 1.5] | 0.787 (0.974) | | -0.17 [-1.9, 1.5] | 0.845 (0.974) | | -0.21 [-1.6, 1.2] | | 0.77 (0.974) |
| **Scale A Foundations of Learning** | | | | | | | | | | | |
| **MMP** | 1 [-1.6, 3.6] | 0.437 (0.974) | -0.87 [-3.9, 2.2] | 0.575 (0.974) | | -1.5 [-4.3, 1.3] | 0.286 (0.974) | | -0.86 [-3.2, 1.5] | | 0.473 (0.974) |
| **MEP** | 1.2 [-0.47, 2.9] | 0.158 (0.974) | -0.064 [-2.6, 2.5] | 0.961 (0.974) | | -0.21 [-2.4, 1.9] | 0.848 (0.974) | | 1.3 [-0.42, 3] | | 0.142 (0.974) |
| **MnBP** | 0.57 [-0.94, 2.1] | 0.462 (0.974) | -0.22 [-1.7, 1.3] | 0.775 (0.974) | | -1.3 [-4, 1.5] | 0.367 (0.974) | | 0.88 [-1.7, 3.5] | | 0.511 (0.974) |
| **MBzP** | 0.81 [-1.4, 3.1] | 0.479 (0.974) | -0.97 [-2.9, 1] | 0.338 (0.974) | | -0.49 [-2.4, 1.4] | 0.619 (0.974) | | 1.1 [-0.85, 3] | | 0.272 (0.974) |
| **ΣDEHP** | 0.33 [-2.3, 2.9] | 0.804 (0.974) | -1 [-3.7, 1.7] | 0.461 (0.974) | | -1.1 [-3.8, 1.6] | 0.422 (0.974) | | 0.61 [-1.6, 2.8] | | 0.59 (0.974) |
| **Scale B Language and Communication** | | | | | | | | | | | |
| **MMP** | -0.43 [-2.3, 1.5] | 0.655 (0.974) | -1.5 [-3.6, 0.57] | 0.157 (0.974) | | -0.4 [-2.3, 1.5] | 0.689 (0.974) | | -0.59 [-2.4, 1.2] | | 0.514 (0.974) |
| **MEP** | 0.099 [-1.1, 1.3] | 0.876 (0.974) | -1.3 [-3, 0.48] | 0.158 (0.974) | | -0.81 [-2.3, 0.66] | 0.283 (0.974) | | -0.22 [-1.5, 1.1] | | 0.749 (0.974) |
| **MnBP** | -0.48 [-1.6, 0.61] | 0.392 (0.974) | -1.3 [-2.3, -0.29] | 0.0136 (0.544) | | -0.28 [-2.2, 1.6] | 0.769 (0.974) | | -0.043 [-2, 1.9] | | 0.966 (0.974) |
| **MBzP** | -0.39 [-2, 1.2] | 0.637 (0.974) | -1.3 [-2.7, 0.012] | 0.0551 (0.974) | | -0.33 [-1.7, 1] | 0.627 (0.974) | | -0.32 [-1.8, 1.2] | | 0.669 (0.974) |
| **ΣDEHP** | -1.2 [-3.1, 0.61] | 0.193 (0.974) | -2.8 [-4.6, -1.1] | 0.00231 (0.256) | | -0.81 [-2.7, 1] | 0.391 (0.974) | | -0.15 [-1.8, 1.5] | | 0.862 (0.974) |
| Scale C Eye and Hand Coordination | | | | | | | | | | | |
| **MMP** | -1.1 [-3, 0.75] | 0.242 (0.974) | -0.13 [-2.4, 2.1] | 0.906 (0.974) | | -0.16 [-2.2, 1.9] | 0.875 (0.974) | | -0.9 [-2.6, 0.8] | | 0.302 (0.974) |
| **MEP** | -0.43 [-1.7, 0.82] | 0.506 (0.974) | -0.16 [-2, 1.7] | 0.865 (0.974) | | -0.71 [-2.3, 0.83] | 0.368 (0.974) | | -1.1 [-2.4, 0.14] | | 0.0855 (0.974) |
| **MnBP** | -0.8 [-1.9, 0.3] | 0.157 (0.974) | -0.45 [-1.5, 0.63] | 0.42 (0.974) | | 1.1 [-0.88, 3.1] | 0.282 (0.974) | | -0.81 [-2.7, 1.1] | | 0.402 (0.974) |
| **MBzP** | -1.3 [-2.9, 0.31] | 0.117 (0.974) | 0.31 [-1.1, 1.8] | 0.676 (0.974) | | 0.35 [-1.1, 1.8] | 0.627 (0.974) | | -0.43 [-1.8, 0.99] | | 0.558 (0.974) |
| **ΣDEHP** | -1.4 [-3.3, 0.45] | 0.139 (0.974) | 0.35 [-1.6, 2.3] | 0.723 (0.974) | | -0.099 [-2.1, 1.9] | 0.921 (0.974) | | -1.1 [-2.7, 0.52] | | 0.189 (0.974) |
| **Scale D Personal-Social-Emotional** | | | | | | | | | | | |
| **MMP** | 0.36 [-1.7, 2.4] | 0.737 (0.974) | -1.8 [-4.1, 0.49] | 0.127 (0.974) | | -0.4 [-2.6, 1.8] | 0.725 (0.974) | | 0.075 [-1.8, 2] | | 0.939 (0.974) |
| **MEP** | 0.35 [-1, 1.7] | 0.612 (0.974) | -0.55 [-2.5, 1.4] | 0.57 (0.974) | | -0.96 [-2.6, 0.7] | 0.262 (0.974) | | -0.13 [-1.6, 1.3] | | 0.859 (0.974) |
| **MnBP** | -1.1 [-2.2, 0.11] | 0.0785 (0.974) | -1.6 [-2.7, -0.53] | 0.00426 (0.256) | | -0.079 [-2.2, 2.1] | 0.943 (0.974) | | 0.75 [-1.4, 2.9] | | 0.49 (0.974) |
| **MBzP** | 0.091 [-1.7, 1.9] | 0.92 (0.974) | -0.041 [-1.5, 1.5] | 0.958 (0.974) | | -0.074 [-1.6, 1.5] | 0.925 (0.974) | | 0.11 [-1.5, 1.7] | | 0.892 (0.974) |
| **ΣDEHP** | -0.34 [-2.4, 1.7] | 0.744 (0.974) | -0.65 [-2.7, 1.4] | 0.525 (0.974) | | -0.88 [-3, 1.2] | 0.415 (0.974) | | 0.11 [-1.7, 1.9] | | 0.907 (0.974) |
| **Scale E Gross Motor** |  |  |  |  | |  |  | |  | |  |
| **MMP** | -0.9 [-3.3, 1.5] | 0.465 (0.974) | -0.27 [-3.1, 2.6] | 0.852 (0.974) | | 0.33 [-2.3, 2.9] | 0.806 (0.974) | | 0.08 [-2.2, 2.3] | | 0.945 (0.974) |
| **MEP** | -0.87 [-2.4, 0.72] | 0.287 (0.974) | -0.78 [-3.2, 1.6] | 0.52 (0.974) | | -1.1 [-3.1, 0.87] | 0.273 (0.974) | | -0.49 [-2.2, 1.2] | | 0.566 (0.974) |
| **MnBP** | -0.81 [-2.2, 0.58] | 0.257 (0.974) | -1.1 [-2.4, 0.29] | 0.125 (0.974) | | 2.5 [0.012, 5] | 0.052 (0.974) | | -0.16 [-2.7, 2.4] | | 0.899 (0.974) |
| **MBzP** | -1.5 [-3.6, 0.58] | 0.162 (0.974) | 0.22 [-1.6, 2] | 0.816 (0.974) | | 0.55 [-1.3, 2.4] | 0.55 (0.974) | | 0.22 [-1.7, 2.1] | | 0.819 (0.974) |
| **ΣDEHP** | -1.3 [-3.7, 1.1] | 0.282 (0.974) | 0.5 [-2.1, 3.1] | 0.702 (0.974) | | 1.8 [-0.65, 4.3] | 0.151 (0.974) | | 0.33 [-1.8, 2.5] | | 0.766 (0.974) |
| ^a^ Results per one unit increase in ln-transformed concentration.  ^b^ Adjusted with Benjamini-Hochberg procedure | | | | | | | |  | |  | |

Table S4: Adjusted linear models between urinary concentrations of phthalate metabolite and parent compound and Griffith Scales of Child Development (GSCD) measured at 6 months in the Modena cohort study, stratified by sampling timing (from mothers close to delivery, M, and in their children at birth, T0, three, T3, and six months, T6.

|  | **M** | | **T0** | | **T3** | | **T6** | |
| --- | --- | --- | --- | --- | --- | --- | --- | --- |
|  | β^a^ [95% CI] | p (p.adj^b^) | β^a^ [95% CI] | p (p.adj^b^) | β^a^ [95% CI] | p (p.adj^b^) | β^a^ [95% CI] | p (p.adj^b^) |
| **General Development Score** | | | | | | | | |
| **MMP** | -0.63 [-2.2, 0.92] | 0.427 (0.907) | -0.43 [-2.3, 1.4] | 0.65 (0.965) | -0.05 [-1.7, 1.6] | 0.952 (0.975) | -0.34 [-1.8, 1.1] | 0.653 (0.965) |
| **MEP** | -0.043 [-1.1, 1] | 0.935 (0.975) | 1.3 [-0.28, 2.8] | 0.112 (0.81) | -0.055 [-1.3, 1.2] | 0.931 (0.975) | 0.14 [-1, 1.3] | 0.81 (0.975) |
| **MnBP** | -0.22 [-1.1, 0.65] | 0.617 (0.965) | -0.05 [-0.99, 0.89] | 0.917 (0.975) | -0.17 [-1.9, 1.6] | 0.851 (0.975) | -0.12 [-1.8, 1.6] | 0.893 (0.975) |
| **MBzP** | -0.82 [-2.1, 0.48] | 0.219 (0.864) | -0.27 [-1.5, 0.92] | 0.653 (0.965) | -0.96 [-2.2, 0.29] | 0.137 (0.81) | -0.54 [-1.9, 0.78] | 0.429 (0.907) |
| **ΣDEHP** | -1 [-2.6, 0.48] | 0.183 (0.813) | -0.55 [-2.2, 1.1] | 0.514 (0.949) | -0.75 [-2.5, 1] | 0.408 (0.907) | -0.38 [-2, 1.2] | 0.647 (0.965) |
| **Scale A Foundations of Learning** | | | | | | | | |
| **MMP** | 0.53 [-2, 3.1] | 0.683 (0.975) | -2.1 [-5.1, 0.83] | 0.162 (0.81) | -1.6 [-4.3, 1.1] | 0.256 (0.884) | -1.6 [-3.9, 0.69] | 0.176 (0.81) |
| **MEP** | 0.48 [-1.2, 2.2] | 0.582 (0.965) | -0.23 [-2.8, 2.4] | 0.862 (0.975) | 0.37 [-1.8, 2.5] | 0.734 (0.975) | 0.31 [-1.5, 2.1] | 0.739 (0.975) |
| **MnBP** | 0.69 [-0.75, 2.1] | 0.348 (0.884) | 0.047 [-1.5, 1.6] | 0.953 (0.975) | -1.1 [-4, 1.8] | 0.45 (0.921) | -0.78 [-3.5, 1.9] | 0.571 (0.965) |
| **MBzP** | 0.82 [-1.3, 3] | 0.457 (0.921) | -2 [-3.9, -0.1] | 0.0424 (0.727) | -0.99 [-3, 1.1] | 0.35 (0.884) | -0.83 [-2.8, 1.2] | 0.422 (0.907) |
| **ΣDEHP** | -0.31 [-2.8, 2.2] | 0.806 (0.975) | -1.3 [-3.9, 1.4] | 0.353 (0.884) | -0.28 [-3.2, 2.7] | 0.854 (0.975) | -0.15 [-2.7, 2.4] | 0.907 (0.975) |
| **Scale B Language and Communication** | | | | | | | | |
| **MMP** | -0.91 [-2.6, 0.81] | 0.302 (0.884) | -0.89 [-2.9, 1.1] | 0.381 (0.907) | 0.28 [-1.5, 2.1] | 0.761 (0.975) | -1.4 [-3, 0.2] | 0.0905 (0.81) |
| **MEP** | -0.065 [-1.2, 1.1] | 0.912 (0.975) | 0.53 [-1.2, 2.3] | 0.549 (0.965) | -0.35 [-1.7, 1] | 0.616 (0.965) | -0.41 [-1.7, 0.88] | 0.536 (0.964) |
| **MnBP** | -0.1 [-1.1, 0.89] | 0.843 (0.975) | -0.27 [-1.3, 0.78] | 0.615 (0.965) | -1.3 [-3.2, 0.49] | 0.157 (0.81) | -1.2 [-3.1, 0.64] | 0.2 (0.827) |
| **MBzP** | -1.1 [-2.5, 0.39] | 0.154 (0.81) | -1.8 [-3, -0.52] | 0.00687 (0.412) | -1.6 [-2.9, -0.28] | 0.0193 (0.579) | -1.7 [-3.1, -0.3] | 0.0191 (0.579) |
| **ΣDEHP** | -1.9 [-3.5, -0.28] | 0.0241 (0.579) | -2.9 [-4.6, -1.3] | 0.000772 (0.0927) | -1.6 [-3.4, 0.29] | 0.103 (0.81) | -1.4 [-3.2, 0.36] | 0.122 (0.81) |
| **Scale C Eye and Hand Coordination** | | | | | | | | |
| **MMP** | -1.3 [-3.2, 0.51] | 0.162 (0.81) | -1.1 [-3.3, 1] | 0.309 (0.884) | -0.21 [-2.2, 1.7] | 0.836 (0.975) | -0.4 [-2.2, 1.4] | 0.654 (0.965) |
| **MEP** | -0.63 [-1.9, 0.61] | 0.321 (0.884) | 0.59 [-1.3, 2.5] | 0.538 (0.964) | -0.77 [-2.3, 0.72] | 0.312 (0.884) | -0.67 [-2, 0.69] | 0.34 (0.884) |
| **MnBP** | -0.77 [-1.8, 0.27] | 0.151 (0.81) | -0.16 [-1.3, 0.97] | 0.787 (0.975) | -0.033 [-2.1, 2] | 0.975 (0.975) | -0.038 [-2.1, 2] | 0.971 (0.975) |
| **MBzP** | -1.5 [-3, 0.047] | 0.0608 (0.81) | -0.2 [-1.6, 1.2] | 0.776 (0.975) | -0.87 [-2.4, 0.61] | 0.251 (0.884) | -0.36 [-1.9, 1.2] | 0.644 (0.965) |
| **ΣDEHP** | -1.3 [-3.1, 0.45] | 0.147 (0.81) | -0.43 [-2.3, 1.5] | 0.66 (0.965) | -1.3 [-3.4, 0.78] | 0.223 (0.864) | -0.64 [-2.6, 1.3] | 0.514 (0.949) |
| **Scale D Personal-Social-Emotional** | | | | | | | | |
| **MMP** | -0.23 [-2, 1.5] | 0.794 (0.975) | -0.75 [-2.8, 1.3] | 0.467 (0.921) | -0.11 [-1.9, 1.7] | 0.905 (0.975) | -0.8 [-2.4, 0.84] | 0.344 (0.884) |
| **MEP** | 0.4 [-0.76, 1.6] | 0.499 (0.949) | 1.8 [0.17, 3.5] | 0.0339 (0.679) | 0.11 [-1.3, 1.5] | 0.877 (0.975) | 0.36 [-0.92, 1.6] | 0.587 (0.965) |
| **MnBP** | -0.42 [-1.4, 0.56] | 0.399 (0.907) | -0.42 [-1.5, 0.61] | 0.424 (0.907) | -0.33 [-2.3, 1.6] | 0.742 (0.975) | 0.11 [-1.8, 2] | 0.91 (0.975) |
| **MBzP** | -0.55 [-2, 0.92] | 0.468 (0.921) | -0.28 [-1.6, 1] | 0.677 (0.975) | -1.1 [-2.5, 0.34] | 0.142 (0.81) | -0.79 [-2.2, 0.6] | 0.27 (0.884) |
| **ΣDEHP** | -1.2 [-2.8, 0.49] | 0.17 (0.81) | -0.89 [-2.7, 0.86] | 0.322 (0.884) | -1.2 [-3.2, 0.79] | 0.239 (0.884) | -0.29 [-2.1, 1.5] | 0.751 (0.975) |
| **Scale E Gross Motor** | | | | | | | | |
| **MMP** | -1.8 [-4.3, 0.73] | 0.169 (0.81) | -0.058 [-3, 2.9] | 0.97 (0.975) | 0.71 [-1.9, 3.4] | 0.6 (0.965) | 0.8 [-1.6, 3.2] | 0.507 (0.949) |
| **MEP** | -1.4 [-3, 0.31] | 0.113 (0.81) | -0.043 [-2.6, 2.5] | 0.974 (0.975) | -1.1 [-3.2, 0.86] | 0.267 (0.884) | -0.085 [-1.9, 1.8] | 0.928 (0.975) |
| **MnBP** | -0.58 [-2, 0.85] | 0.431 (0.907) | -0.77 [-2.3, 0.76] | 0.326 (0.884) | 1.9 [-0.9, 4.6] | 0.19 (0.813) | 0.5 [-2.3, 3.3] | 0.721 (0.975) |
| **MBzP** | -1.8 [-3.9, 0.31] | 0.098 (0.81) | -0.31 [-2.2, 1.6] | 0.753 (0.975) | -0.16 [-2.2, 1.9] | 0.876 (0.975) | 0.43 [-1.7, 2.6] | 0.695 (0.975) |
| **ΣDEHP** | -1.9 [-4.4, 0.61] | 0.143 (0.81) | -0.34 [-3, 2.3] | 0.805 (0.975) | 1.4 [-1.4, 4.2] | 0.334 (0.884) | 1.2 [-1.4, 3.8] | 0.373 (0.907) |

^a^ Results per one unit increase in ln-transformed concentration.
^b^ Adjusted with Benjamini-Hochberg procedure

*Model adjusted for gestational age at delivery, delivery mode, maternal age , mother’s educational level, father’s educational level, pre-gravidic BMI and if GSCD was performed before or after the pandemic outbreak.*

Table S5: Female adjusted linear models between urinary concentrations of phthalate metabolite and parent compound and Griffith Scales of Child Development (GSCD) measured at 6 months in the Modena cohort study, stratified by sampling timing (from mothers close to delivery, M, and in their children at birth, T0, three, T3, and six months, T6.

| **FEMALE** |  | |  | | |  | |  | |
| --- | --- | --- | --- | --- | --- | --- | --- | --- | --- |
|  | **M** | | **T0** | | | **T3** | | **T6** | |
|  | β^a^ [95% CI] | p (p.adj^b^) | | β^a^ [95% CI] | p (p.adj^b^) | β^a^ [95% CI] | p (p.adj^b^) | β^a^ [95% CI] | p (p.adj^b^) |
| **General Development Score** | |  | |  |  |  |  |  |  |
| **MMP** | -0.62 [-2.7, 1.5] | 0.571 (0.996) | | 1.2 [-1.2, 3.6] | 0.34 (0.996) | 1 [-0.81, 2.8] | 0.287 (0.996) | -0.095 [-2, 1.8] | 0.925 (0.996) |
| **MEP** | 0.17 [-1.1, 1.4] | 0.795 (0.996) | | 2.8 [0.87, 4.8] | 0.00935 (0.561) | 0.24 [-1.2, 1.7] | 0.756 (0.996) | 0.8 [-0.46, 2.1] | 0.222 (0.996) |
| **MnBP** | -0.33 [-1.5, 0.85] | 0.591 (0.996) | | 0.47 [-0.92, 1.9] | 0.514 (0.996) | 0.19 [-1.6, 2] | 0.84 (0.996) | 0.33 [-1.7, 2.4] | 0.757 (0.996) |
| **MBzP** | -0.33 [-2.2, 1.5] | 0.728 (0.996) | | 0.81 [-0.96, 2.6] | 0.379 (0.996) | 0.21 [-1.5, 1.9] | 0.815 (0.996) | -0.78 [-2.4, 0.82] | 0.346 (0.996) |
| **ΣDEHP** | -1.1 [-3.1, 1] | 0.322 (0.996) | | -0.35 [-2.5, 1.8] | 0.752 (0.996) | -1.3 [-3.3, 0.73] | 0.224 (0.996) | -0.66 [-2.6, 1.2] | 0.499 (0.996) |
| **Scale A Foundations of Learning** | | |  | |  |  |  |  |  |
| **MMP** | 3.7 [-0.29, 7.7] | 0.0786 (0.996) | | -0.25 [-5.4, 4.9] | 0.925 (0.996) | -0.38 [-4.3, 3.5] | 0.847 (0.996) | -1.3 [-4.9, 2.4] | 0.506 (0.996) |
| **MEP** | 1.6 [-0.9, 4.1] | 0.221 (0.996) | | 4.4 [-0.1, 8.9] | 0.0673 (0.996) | -0.52 [-3.6, 2.5] | 0.742 (0.996) | 0.0065 [-2.4, 2.5] | 0.996 (0.996) |
| **MnBP** | 1.1 [-1.2, 3.5] | 0.353 (0.996) | | 0.43 [-2.6, 3.4] | 0.781 (0.996) | -1.2 [-5.1, 2.6] | 0.531 (0.996) | -0.38 [-4.3, 3.6] | 0.85 (0.996) |
| **MBzP** | 3 [-0.64, 6.6] | 0.117 (0.996) | | -1.9 [-5.5, 1.8] | 0.33 (0.996) | 1.4 [-2, 4.9] | 0.416 (0.996) | 0.31 [-2.9, 3.5] | 0.851 (0.996) |
| **ΣDEHP** | 2.2 [-1.9, 6.4] | 0.296 (0.996) | | 0.44 [-4.1, 4.9] | 0.849 (0.996) | -0.092 [-4.4, 4.2] | 0.966 (0.996) | 0.17 [-3.6, 4] | 0.93 (0.996) |
| **Scale B Language and Communication** | | |  | |  |  |  |  |  |
| **MMP** | -0.6 [-2.8, 1.6] | 0.601 (0.996) | | 1.3 [-1.1, 3.8] | 0.301 (0.996) | 1.3 [-0.51, 3.1] | 0.17 (0.996) | -1.5 [-3.4, 0.34] | 0.119 (0.996) |
| **MEP** | 0.063 [-1.3, 1.4] | 0.928 (0.996) | | 2.5 [0.39, 4.7] | 0.0292 (0.876) | 0.21 [-1.3, 1.7] | 0.789 (0.996) | -0.31 [-1.6, 0.99] | 0.643 (0.996) |
| **MnBP** | -0.62 [-1.9, 0.63] | 0.336 (0.996) | | 0.31 [-1.2, 1.8] | 0.68 (0.996) | 0.27 [-1.6, 2.1] | 0.783 (0.996) | -0.51 [-2.6, 1.6] | 0.637 (0.996) |
| **MBzP** | -0.72 [-2.7, 1.3] | 0.482 (0.996) | | -0.73 [-2.5, 1.1] | 0.44 (0.996) | -0.015 [-1.7, 1.7] | 0.987 (0.996) | -0.68 [-2.4, 1] | 0.439 (0.996) |
| **ΣDEHP** | -0.94 [-3.2, 1.3] | 0.411 (0.996) | | -1.7 [-3.8, 0.44] | 0.134 (0.996) | -0.79 [-2.8, 1.3] | 0.453 (0.996) | -1.7 [-3.6, 0.24] | 0.0969 (0.996) |
| **Scale C Eye and Hand Coordination** | | |  | |  |  |  |  |  |
| **MMP** | 0.045 [-2.5, 2.6] | 0.972 (0.996) | | 1.6 [-1.4, 4.6] | 0.307 (0.996) | 1.6 [-0.57, 3.7] | 0.163 (0.996) | -0.64 [-2.9, 1.6] | 0.582 (0.996) |
| **MEP** | -0.58 [-2.1, 0.94] | 0.46 (0.996) | | 1.2 [-1.6, 4] | 0.409 (0.996) | 1.2 [-0.47, 2.9] | 0.168 (0.996) | 0.11 [-1.4, 1.6] | 0.884 (0.996) |
| **MnBP** | -0.5 [-1.9, 0.93] | 0.496 (0.996) | | 0.9 [-0.85, 2.6] | 0.325 (0.996) | 1.1 [-1.1, 3.3] | 0.324 (0.996) | -0.28 [-2.7, 2.2] | 0.825 (0.996) |
| **MBzP** | 0.13 [-2.1, 2.4] | 0.911 (0.996) | | 2 [-0.11, 4.1] | 0.0754 (0.996) | 1.1 [-0.87, 3] | 0.29 (0.996) | -0.72 [-2.7, 1.3] | 0.484 (0.996) |
| **ΣDEHP** | 0.49 [-2.1, 3] | 0.71 (0.996) | | -0.35 [-3, 2.3] | 0.799 (0.996) | 0.014 [-2.4, 2.4] | 0.991 (0.996) | -1.3 [-3.6, 0.98] | 0.27 (0.996) |
| **Scale D Personal-Social-Emotional** | | |  | |  |  |  |  |  |
| **MMP** | -0.18 [-3.1, 2.7] | 0.906 (0.996) | | 1.6 [-1.6, 4.9] | 0.337 (0.996) | 0.78 [-1.8, 3.3] | 0.559 (0.996) | -0.94 [-3.6, 1.7] | 0.491 (0.996) |
| **MEP** | 0.83 [-0.87, 2.5] | 0.345 (0.996) | | 4.8 [2.4, 7.2] | 0.000695 (0.0834) | 0.18 [-1.9, 2.3] | 0.865 (0.996) | 1.3 [-0.41, 3] | 0.148 (0.996) |
| **MnBP** | -0.064 [-1.7, 1.6] | 0.939 (0.996) | | 0.44 [-1.5, 2.4] | 0.658 (0.996) | 0.08 [-2.5, 2.7] | 0.952 (0.996) | 0.81 [-2, 3.6] | 0.576 (0.996) |
| **MBzP** | -0.45 [-3, 2.1] | 0.729 (0.996) | | 1.2 [-1.2, 3.7] | 0.321 (0.996) | 0.4 [-2, 2.8] | 0.742 (0.996) | -0.86 [-3.1, 1.4] | 0.457 (0.996) |
| **ΣDEHP** | -0.91 [-3.8, 2] | 0.54 (0.996) | | -0.59 [-3.5, 2.3] | 0.694 (0.996) | -1.5 [-4.3, 1.4] | 0.318 (0.996) | 0.61 [-2, 3.2] | 0.652 (0.996) |
| **Scale E Gross Motor** | |  | |  |  |  |  |  |  |
| **MMP** | -2.8 [-6.7, 1] | 0.159 (0.996) | | 0.16 [-4.3, 4.6] | 0.945 (0.996) | -0.15 [-3.6, 3.3] | 0.932 (0.996) | 0.69 [-2.9, 4.3] | 0.707 (0.996) |
| **MEP** | -0.68 [-3.1, 1.7] | 0.579 (0.996) | | 0.9 [-3.2, 5] | 0.67 (0.996) | -1.7 [-4.3, 0.96] | 0.223 (0.996) | 0.067 [-2.3, 2.5] | 0.956 (0.996) |
| **MnBP** | -1.3 [-3.5, 0.89] | 0.251 (0.996) | | -1.1 [-3.6, 1.5] | 0.421 (0.996) | 0.98 [-2.4, 4.4] | 0.577 (0.996) | 1.5 [-2.3, 5.4] | 0.434 (0.996) |
| **MBzP** | -2.2 [-5.7, 1.3] | 0.223 (0.996) | | -1.7 [-4.8, 1.5] | 0.307 (0.996) | 0.47 [-2.6, 3.5] | 0.768 (0.996) | -0.14 [-3.3, 3] | 0.929 (0.996) |
| **ΣDEHP** | -4.3 [-8, -0.62] | 0.029 (0.876) | | -0.72 [-4.6, 3.1] | 0.715 (0.996) | -0.12 [-3.9, 3.7] | 0.951 (0.996) | 0.66 [-3.1, 4.4] | 0.73 (0.996) |

^a^ Results per one unit increase in log-transformed concentration
^b^ Adjusted with Benjamini-Hochberg procedure

*Model adjusted for gestational age at delivery, delivery mode, maternal age , mother’s educational level, father’s educational level, pre-gravidic BMI and if GSCD was performed before or after the pandemic outbreak.*

Table S6: Male adjusted linear models between urinary concentrations of phthalate metabolite and parent compound and Griffith Scales of Child Development (GSCD) measured at 6 months in the Modena cohort study, stratified by sampling timing (from mothers close to delivery, M, and in their children at birth, T0, three, T3, and six months, T6.

| **MALE** |  | |  | |  | |  | |
| --- | --- | --- | --- | --- | --- | --- | --- | --- |
|  | **M** | | **T0** | | **T3** | | **T6** | |
|  | β^a^ [95% CI] | p (p.adj^b^) | β^a^ [95% CI] | p (p.adj^b^) | β^a^ [95% CI] | p (p.adj^b^) | β^a^ [95% CI] | p (p.adj^b^) |
| **General Development Score** | |  |  |  |  |  |  |  |
| **MMP** | -0.39 [-2.6, 1.9] | 0.736 (0.959) | -1.6 [-4.1, 0.91] | 0.221 (0.889) | -0.12 [-2.6, 2.3] | 0.926 (0.98) | -0.31 [-2.4, 1.7] | 0.767 (0.959) |
| **MEP** | -0.12 [-1.6, 1.4] | 0.877 (0.975) | 0.72 [-1.5, 2.9] | 0.528 (0.959) | -0.18 [-2.1, 1.7] | 0.858 (0.975) | -0.34 [-2.1, 1.4] | 0.711 (0.959) |
| **MnBP** | -0.14 [-1.3, 1] | 0.821 (0.962) | -0.27 [-1.5, 0.91] | 0.654 (0.959) | -0.21 [-3.6, 3.2] | 0.904 (0.98) | 0.42 [-2.2, 3.1] | 0.755 (0.959) |
| **MBzP** | -0.53 [-2.2, 1.2] | 0.546 (0.959) | -0.46 [-2, 1.1] | 0.562 (0.959) | -1.1 [-2.9, 0.6] | 0.207 (0.889) | 0.35 [-1.7, 2.4] | 0.734 (0.959) |
| **ΣDEHP** | -0.66 [-2.7, 1.4] | 0.53 (0.959) | -0.57 [-2.9, 1.7] | 0.627 (0.959) | 0.37 [-2.4, 3.2] | 0.798 (0.962) | 0.45 [-2, 2.9] | 0.715 (0.959) |
| **Scale A Foundations of Learning** | |  |  |  |  |  |  |  |
| **MMP** | 0.16 [-3.3, 3.6] | 0.93 (0.98) | -2.4 [-6.2, 1.3] | 0.214 (0.889) | -3.2 [-6.9, 0.42] | 0.0906 (0.838) | -1.4 [-4.4, 1.6] | 0.366 (0.959) |
| **MEP** | 0.53 [-1.7, 2.8] | 0.642 (0.959) | -1.4 [-4.8, 2] | 0.414 (0.959) | 0.13 [-2.9, 3.1] | 0.933 (0.98) | 0.73 [-1.9, 3.3] | 0.585 (0.959) |
| **MnBP** | 0.71 [-1.1, 2.5] | 0.444 (0.959) | 0.25 [-1.6, 2.1] | 0.795 (0.962) | -5.1 [-9.8, -0.44] | 0.038 (0.838) | 0.26 [-3.6, 4.1] | 0.895 (0.98) |
| **MBzP** | 0.31 [-2.3, 2.9] | 0.82 (0.962) | -1.4 [-3.7, 0.84] | 0.222 (0.889) | -2.6 [-5.2, -0.056] | 0.052 (0.838) | -0.55 [-3.3, 2.2] | 0.698 (0.959) |
| **ΣDEHP** | -0.39 [-3.5, 2.7] | 0.802 (0.962) | -0.61 [-4, 2.7] | 0.723 (0.959) | -2.1 [-6.4, 2.1] | 0.324 (0.959) | 0.71 [-2.8, 4.2] | 0.693 (0.959) |
| **Scale B Language and Communication** | |  |  |  |  |  |  |  |
| **MMP** | -1.3 [-4, 1.4] | 0.344 (0.959) | -2.3 [-5.2, 0.54] | 0.119 (0.838) | 0.0096 [-2.9, 2.9] | 0.995 (0.999) | -1 [-3.4, 1.4] | 0.412 (0.959) |
| **MEP** | -0.0016 [-1.7, 1.7] | 0.999 (0.999) | 0.73 [-1.9, 3.4] | 0.592 (0.959) | -0.65 [-2.9, 1.6] | 0.575 (0.959) | -0.43 [-2.5, 1.7] | 0.695 (0.959) |
| **MnBP** | 0.38 [-1, 1.8] | 0.597 (0.959) | -0.28 [-1.7, 1.1] | 0.704 (0.959) | -3.2 [-6.8, 0.35] | 0.0849 (0.838) | -0.73 [-3.8, 2.4] | 0.647 (0.959) |
| **MBzP** | -1 [-3.1, 1] | 0.324 (0.959) | -2.2 [-3.8, -0.48] | 0.0156 (0.838) | -2.1 [-4, -0.17] | 0.0392 (0.838) | -1.8 [-4, 0.34] | 0.105 (0.838) |
| **ΣDEHP** | -2 [-4.3, 0.29] | 0.0929 (0.838) | -3.6 [-6, -1.2] | 0.0048 (0.576) | -2.2 [-5.3, 0.94] | 0.178 (0.889) | -0.54 [-3.4, 2.3] | 0.713 (0.959) |
| **Scale C Eye and Hand Coordination** | |  |  |  |  |  |  |  |
| **MMP** | -1.8 [-4.6, 0.93] | 0.2 (0.889) | -3 [-6, -0.052] | 0.0524 (0.838) | -0.53 [-3.6, 2.6] | 0.737 (0.959) | -0.48 [-3, 2.1] | 0.714 (0.959) |
| **MEP** | -0.62 [-2.5, 1.2] | 0.51 (0.959) | 0.15 [-2.6, 2.9] | 0.917 (0.98) | -2 [-4.3, 0.37] | 0.108 (0.838) | -1.1 [-3.3, 1.1] | 0.316 (0.959) |
| **MnBP** | -0.84 [-2.3, 0.62] | 0.266 (0.941) | -0.83 [-2.3, 0.62] | 0.269 (0.941) | -0.92 [-5.1, 3.3] | 0.674 (0.959) | 0.51 [-2.7, 3.8] | 0.762 (0.959) |
| **MBzP** | -1.6 [-3.7, 0.48] | 0.137 (0.878) | -1.2 [-3, 0.6] | 0.194 (0.889) | -1.5 [-3.7, 0.69] | 0.188 (0.889) | -0.046 [-2.4, 2.3] | 0.97 (0.995) |
| **ΣDEHP** | -2 [-4.4, 0.45] | 0.118 (0.838) | -1.3 [-4, 1.4] | 0.342 (0.959) | -1.6 [-5.1, 1.9] | 0.374 (0.959) | -0.12 [-3.1, 2.9] | 0.939 (0.98) |
| **Scale D Personal-Social-Emotional** | |  |  |  |  |  |  |  |
| **MMP** | -0.39 [-2.8, 2] | 0.754 (0.959) | -3.1 [-5.6, -0.54] | 0.022 (0.838) | 0.013 [-2.7, 2.7] | 0.993 (0.999) | -0.85 [-3, 1.3] | 0.434 (0.959) |
| **MEP** | -0.16 [-1.7, 1.4] | 0.846 (0.975) | 0.42 [-2, 2.8] | 0.732 (0.959) | 0.27 [-1.8, 2.4] | 0.803 (0.962) | -0.57 [-2.4, 1.3] | 0.547 (0.959) |
| **MnBP** | -0.62 [-1.9, 0.62] | 0.332 (0.959) | -0.96 [-2.2, 0.29] | 0.139 (0.878) | -0.12 [-3.8, 3.5] | 0.95 (0.982) | -0.31 [-3, 2.4] | 0.826 (0.962) |
| **MBzP** | -0.32 [-2.2, 1.5] | 0.73 (0.959) | -0.95 [-2.5, 0.64] | 0.248 (0.941) | -1.6 [-3.5, 0.22] | 0.0926 (0.838) | -1.1 [-3.1, 0.81] | 0.261 (0.941) |
| **ΣDEHP** | -1.7 [-3.8, 0.33] | 0.107 (0.838) | -1.9 [-4.2, 0.36] | 0.107 (0.838) | -0.52 [-3.6, 2.5] | 0.738 (0.959) | -0.89 [-3.3, 1.6] | 0.482 (0.959) |
| **Scale E Gross Motor** | |  |  |  |  |  |  |  |
| **MMP** | -0.64 [-4.2, 2.9] | 0.723 (0.959) | 0.94 [-3, 4.9] | 0.645 (0.959) | 1.8 [-2.1, 5.7] | 0.369 (0.959) | 0.87 [-2.3, 4] | 0.593 (0.959) |
| **MEP** | -1.5 [-3.8, 0.75] | 0.195 (0.889) | -0.57 [-4, 2.9] | 0.747 (0.959) | -2 [-4.9, 1] | 0.203 (0.889) | -0.42 [-3.1, 2.3] | 0.764 (0.959) |
| **MnBP** | -0.15 [-2, 1.7] | 0.876 (0.975) | -0.49 [-2.4, 1.4] | 0.604 (0.959) | 1.8 [-3.6, 7.1] | 0.523 (0.959) | 0.32 [-3.7, 4.4] | 0.878 (0.975) |
| **MBzP** | -1.2 [-3.9, 1.5] | 0.381 (0.959) | 0.78 [-1.6, 3.2] | 0.526 (0.959) | -0.5 [-3.3, 2.3] | 0.728 (0.959) | 1.2 [-1.9, 4.3] | 0.461 (0.959) |
| **ΣDEHP** | -0.56 [-3.8, 2.7] | 0.735 (0.959) | 0.97 [-2.6, 4.5] | 0.599 (0.959) | 2.8 [-1.6, 7.1] | 0.222 (0.889) | 2.1 [-1.6, 5.7] | 0.275 (0.941) |

^a^ Results per one unit increase in ln-transformed concentration
^b^ Adjusted with Benjamini-Hochberg procedure

*Model adjusted for gestational age at delivery, delivery mode, maternal age , mother’s educational level, father’s educational level, pre-gravidic BMI and if GSCD was performed before or after the pandemic outbreak.*
